# Responses of several measures to different intensity levels of upper limb exergames in children with neurological diagnoses: a pilot study

**DOI:** 10.3389/fresc.2024.1405304

**Published:** 2024-10-23

**Authors:** Gaizka Goikoetxea-Sotelo, Hubertus J. A. van Hedel

**Affiliations:** ^1^ Swiss Children’s Rehab, University Children’s Hospital Zurich, University of Zurich, Affoltern am Albis, Switzerland; ^2^ Children’s Research Center, University Children’s Hospital Zurich, University of Zurich, Zurich, Switzerland; ^3^ Department of Health Sciences and Technology, ETH Zurich, Zurich, Switzerland

**Keywords:** intensity measures, neurorehabilitation, movement repetitions, heart rate variability, activity counts, skin conductance, borg scale

## Abstract

**Background:**

Therapy intensity is among the most critical factors influencing neurorehabilitative outcomes. Because of its simplicity, time spent in therapy is the most commonly used measure of therapy intensity. However, time spent in therapy is only a vague estimate of how hard a patient works during therapy. Several measures have been proposed to better capture the amount of work a patient puts forth during therapy. Still, it has never been analyzed how these measures respond to changes in therapist-selected exercise intensity in children with neurological conditions.

**Objectives:**

To investigate the response and the reliability of heart rate variability (HRV), skin conductance (SC), activity counts per minute (AC/min), movement repetitions per minute (MOV/min), and perceived exertion to different therapist-tailored intensity levels of upper limb technology-assisted therapy in children with neurological conditions.

**Methods:**

In this pilot cross-sectional study, participants engaged in three personalized, randomized exergame intensity levels (“very easy”, “challenging”, “very difficult”) for eight minutes each. We assessed all measures at each intensity level. The experiment was conducted twice on two consecutive days. We quantified reliability using intra-class correlation coefficients (ICC).

**Results:**

We included 12 children and adolescents aged 11.92 (±3.03) years. HRV, MOV/min, and perceived exertion could differentiate among the three intensity levels. HRV, MOV/min, perceived exertion, and AC/min showed moderate to excellent (0.62 ≤ ICC ≤ 0.98) test-retest reliability.

**Conclusion:**

HRV, MOV/min, and perceived exertion show potential for becoming valid and reliable intensity measures for an upper limb robotic rehabilitative setting. However, studies with larger sample sizes and more standardized approaches are needed to understand these measures’ responses better.

## 1 Introduction

Neurorehabilitation aims at improving a patient's level of functioning within their usual environment (1). With this goal in mind, patients and therapists work together to treat specific impairments in the body structures and functions and learn new strategies to overcome limitations in activities of daily living. The best evidence for the improvement of functions (in any neurological condition) suggests that therapy should involve exercises that are highly intensive, repetitive, goal-directed, and task-specific (2–6), which is in line with motor learning theories (7). However, while assessing whether an exercise is repetitive, goal-directed, and task-specific seems straightforward, measuring the intensity, i.e., the amount of physical or mental work put forth by the patient during a particular movement or series of movements, exercise, or activity (8), appears problematic. As intensity is one of the most critical factors affecting rehabilitative outcomes (9), it is reasonable to suggest that we should improve its quantification. Valid and reliable intensity measures could improve our estimations of the minimal amount of therapy needed to improve functional outcomes and help compare the effectiveness of different therapy approaches while controlling for the amount of therapy.

Time spent in therapy remains the most commonly used measure of therapy intensity (10). However, time spent in treatment is only a vague estimate of how hard a patient works during therapy (11). Although active therapy time has emerged as a better representative of the amount of exercise a patient performs during a therapy session, it still does not give information about how hard the patient exercises. Other measures, such as the number of movement repetitions or activity counts, have been suggested to quantify what a patient does during therapy or daily life (12, 13). Although they are better representatives of what a patient does during therapy than time spent in therapy or active time, as they account for rest periods, they still fail to inform us about how hard the patient engages in the movement. However, by adding a temporal constraint to the measures, i.e., calculating the number of movement repetitions per minute (MOV/min) or activity counts per minute (AC/min), it is possible to get a better indication of how hard a patient works, i.e., intensity. For instance, performing 60 movement repetitions of a specific exercise in one minute reflects a higher intensity than performing 60 repetitions in five minutes. Nevertheless, these measures do not account for other important aspects of therapy intensity, such as motor and mental task complexity.

Self-reported perceived exertion scales, such as the adapted Borg scale (14), have proved useful for grading the training intensity of upper limb functional training programs in adult stroke patients without cognitive impairments (15), and, therefore, could reflect exercise intensity at a more complex and multidimensional level. However, patients with neurological diagnoses, especially children, often experience impairments in cognition and comprehension, which may interfere with the correct use of these questionnaires.

Therefore, it seems reasonable to suggest that valid, objective, and more accurate measures are needed to quantify upper limb motor learning-based therapy intensity (16). In this context, physiological measures such as heart rate variability (HRV) and skin conductance (SC) have the potential to provide a more accurate and reliable assessment of therapy intensity. Heart rate variability and SC reflect heart-brain interaction and central nervous system (CNS) modulation, which are affected by physical and cognitive complex tasks or stressful situations (17–19).

Heart rate variability is an indirect marker reflecting the interplay between the autonomic nervous system, comprising the sympathetic and parasympathetic branches, and the CNS. Under the influence of the CNS, the autonomic nervous system regulates the heart's activity, with the sympathetic branch typically increasing the heart rate and the parasympathetic branch decreasing it. Heart rate variability measures the variation in time intervals between consecutive heartbeats, providing insights into the dynamic balance and flexibility of the autonomic nervous system (20). Higher HRV often signifies greater adaptability and regulatory capacity of the CNS over physiological processes, which is usually visible in physiologically relaxed or non-stressful situations, while reduced HRV may suggest a less adaptable and potentially compromised neural regulatory system, usually seen in physiologically demanding (i.e., physically or cognitively) or stressful situations (21). Because of these characteristics, changes in HRV profiles have been found in studies analyzing the effects of increments in mental task difficulty (17, 18) and motor intensity (22, 23) in healthy adults. Therefore, this leads us to assume that HRV could indicate therapy intensity in pediatric upper limb neurorehabilitation, with lower values indicating higher intensity.

Skin conductance (SC) reflects the autonomic nervous system's activity, specifically the sympathetic branch. Changes in SC occur due to variations in sweat gland activity, controlled by the sympathetic nervous system. Heightened conductance often accompanies increased sympathetic arousal, reflecting the body's response to emotional, cognitive, or physiological stimuli (24). Skin conductance reacts to changes in task difficulty during video gaming (18, 19, 25, 26), which grants it potential as an intensity measure in pediatric upper limb neurorehabilitation, with higher therapy intensities eliciting higher SC.

In this first exploratory study, we assessed how HRV, SC, AC/min, MOV/min, and perceived exertion respond to three different therapist-tailored intensity levels of upper limb robotic exercises in children with neurological diagnoses. We hypothesized that (1) HRV would be lower at higher therapy intensity levels than at lower levels, and (2) SC, AC/min, MOV/min, and perceived exertion would be higher at higher therapy intensity levels than at lower levels. Furthermore, we investigated the test-retest reliability of the measures. We deem this necessary because (1) to be of use to adapt and individualize therapy, intensity measures should also be reliable, and (2) although the reliability of most of these measures has already been assessed before (27–30), it has never been assessed in our target population during active exercise. We consider an intraclass correlation coefficient (ICC) exceeding 0.75 acceptable.

## 2 Methods

### 2.1 Participants

The goal was to recruit 18 children and adolescents with neurological conditions, distributed equally across the age groups 5–8 years, 9–12 years, and 13–18 years, in line with Jean Piaget’s theories of cognitive development (31), covering all the cognitive developmental stages.

Recruitment was performed at the Swiss Children's Rehab. Inclusion criteria were: age between 5 and 18 years, neurological diagnoses affecting the upper limbs, ability to understand easy instructions, being able to communicate discomfort or pain, absence of screen-triggered epilepsy, and intact skin on the locations where we had to position the sensors for measuring the heart rate or SC. Additionally, the children had to receive upper limb robotic therapy and show compliance. We derived information on the age, sex, diagnosis, weight, and height from the electronic records from our hospital's intern database.

Eligible participants and their legal representative(s) were informed about the study verbally and also in writing for those aged ten years and older. All participants and parents had to provide verbal consent. In addition, we obtained written consent from at least one legal representative and participants aged 14 years and above. The Ethics Committee of the Canton of Zurich reviewed the study protocol (BASEC Nr. Req-2021-00826). We performed the study in line with the Declaration of Helsinki and Good Clinical Practice guidelines.

### 2.2 Rehabilitation technologies

We used two CE-certified rehabilitation therapy technologies during the experiment: the Myro® (Tyromotion GmbH, Graz, Austria) and the Armeo Spring Pediatric® (Hocoma AG, Volketswil, Switzerland). These devices allowed us to include patients with different severity levels.

**Figure 1:**
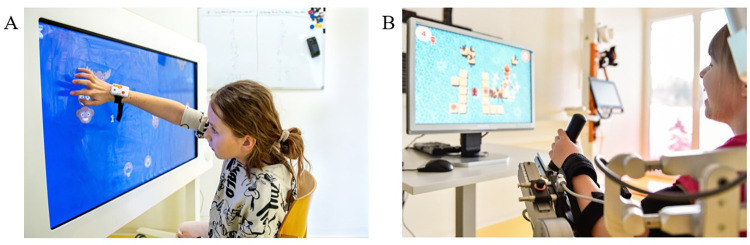
Rehabilitation technologies. Two patients performing technology-assisted upper limb therapies in the **(A)** Myro® (Tyromotion GmbH, Graz, Austria) and **(B)** Armeo Spring Pediatric® (Hocoma AG, Volketswil, Switzerland) devices.

The Myro® (Figure 1A) is a touch screen device that enables, among other things, the training of gross and fine motor skills through video gaming without providing physical support. It can react both to motion and pressure. The therapist can adapt the Myro® to the patient's need by adjusting the angulation, height, and work surface. These were set in advance for each patient and kept unaltered during the experimental procedure.

The Armeo Spring Pediatric® (Figure 1B) is a unilateral exoskeleton device that supports the weight of the upper and lower arm mechanically (using springs), enabling patients to interact with the 3D environment. The therapist can adjust the device to support the right or left arm and adjust the shoulder height and upper and lower arm lengths to the participant's anthropometry. It has 6 degrees of freedom and allows the following ranges of motion (shoulder adduction/abduction −169° to +50°, flexion/extension +40° to +120°, and internal/external rotation 0° to 90°; elbow flexion/extension 0° to 100°; forearm pronation/supination −60° to 60°; and wrist flexion/extension −60° to 60°). The device also has a grip pressure sensor in the hand module. The device settings were individually adapted for each patient before starting the procedure and kept unaltered during the experimental procedure.

### 2.3 Procedures

We collected the data between September 2021 and November 2022. In short, the participants played the exergames on the same upper extremity rehabilitative devices they used during regular therapy. They engaged in three different exergame intensity levels (“very easy”, “challenging”, and “very difficult”) for 8 min each, while several measures captured the participants’ responses. The patient's primary therapist tailored these intensity levels according to the patient's capabilities and therapy goals. The total duration of the session was around 60 min. We administered the same measurement protocol on two consecutive days to explore the test-retest reliability.

Before starting the study, we explained the procedures and protocols to the participants. As the participants were already familiar with the devices, no familiarization protocol was required. The study was conducted in the same room and at the first available therapy slot (8:00 a.m.) to avoid interferences from previously performed therapies or activities.

**Figure 2:**
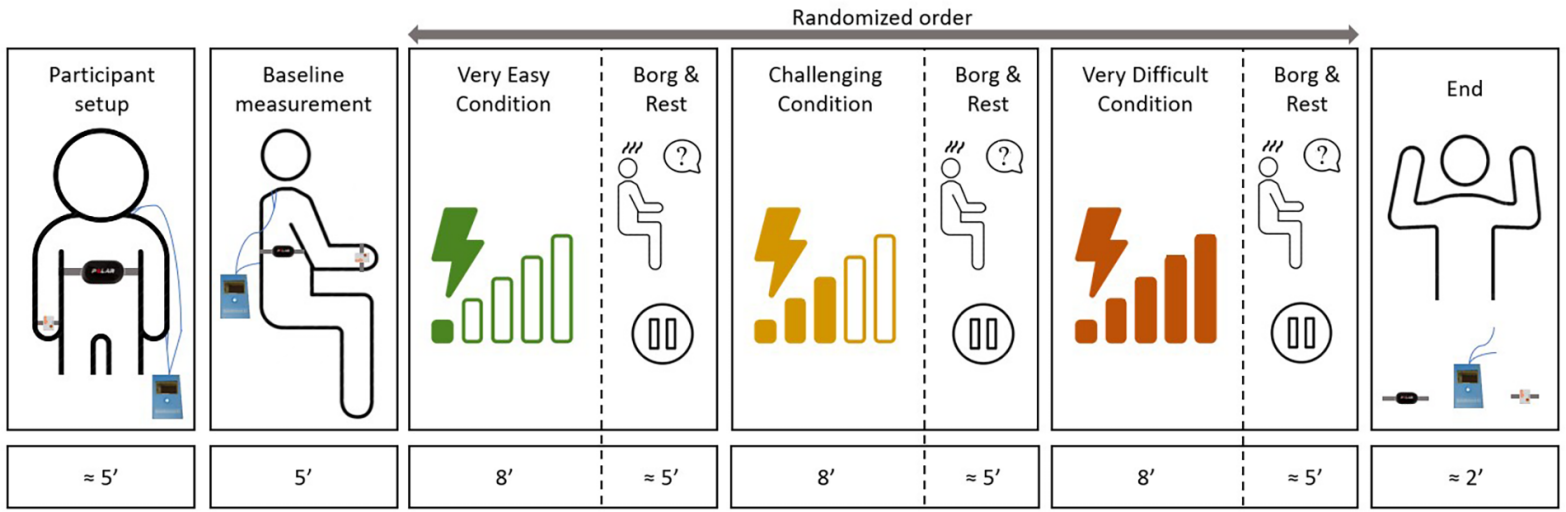
Timeline of the experimental procedures. The study protocol consisted a baseline measure and three individually adapted, randomized intensity levels. The complete protocol lasted around one hour for each patient.

After explaining the procedures, the therapist attached the Polar chest strap to measure the heart rate, the electrodes to measure SC, and the inertial measurement unit (IMU) to measure movement-based data (Figure 2). We positioned a camera behind the child to record the interactions with the rehabilitation technology. We turned all measurement devices on and off simultaneously (i.e., at the beginning and at the end of the session) to synchronize the data. Before starting with the intensity levels, we performed a 5-min baseline measurement.

The therapist set the “challenging” level based on what the patient had been doing in therapy at the time of the experiment. Based on the “challenging” level, the therapist defined the “very easy” and “very difficult” levels. We asked the therapists to aim that the participants would get near 100% success rates without effort in the “very easy” level. In the “very difficult” level, the participants would get success rates under 60% with maximum effort. We did not standardize how therapists had to adapt the intensity levels, as the patients’ capabilities and goals differed significantly. We suggested the therapists to adapt the intensity in the same way they would have done it during therapy and to try to keep the intensity constant for the 8-minute period. This helped us keep the experiment close to the clinical practice, which would improve the interpretation of the results. The intensity levels from the first session were logged and repeated, in the same order, during the second session (the selected games and levels can be found under Supplementary Material S1).

The participant played each exergame at the corresponding intensity for eight minutes. After each intensity level, the children rated their perceived level of exertion. After that, we granted a rest-as-needed period, which usually lasted around 5 min.

### 2.4 Intensity measures

**Figure 3:**
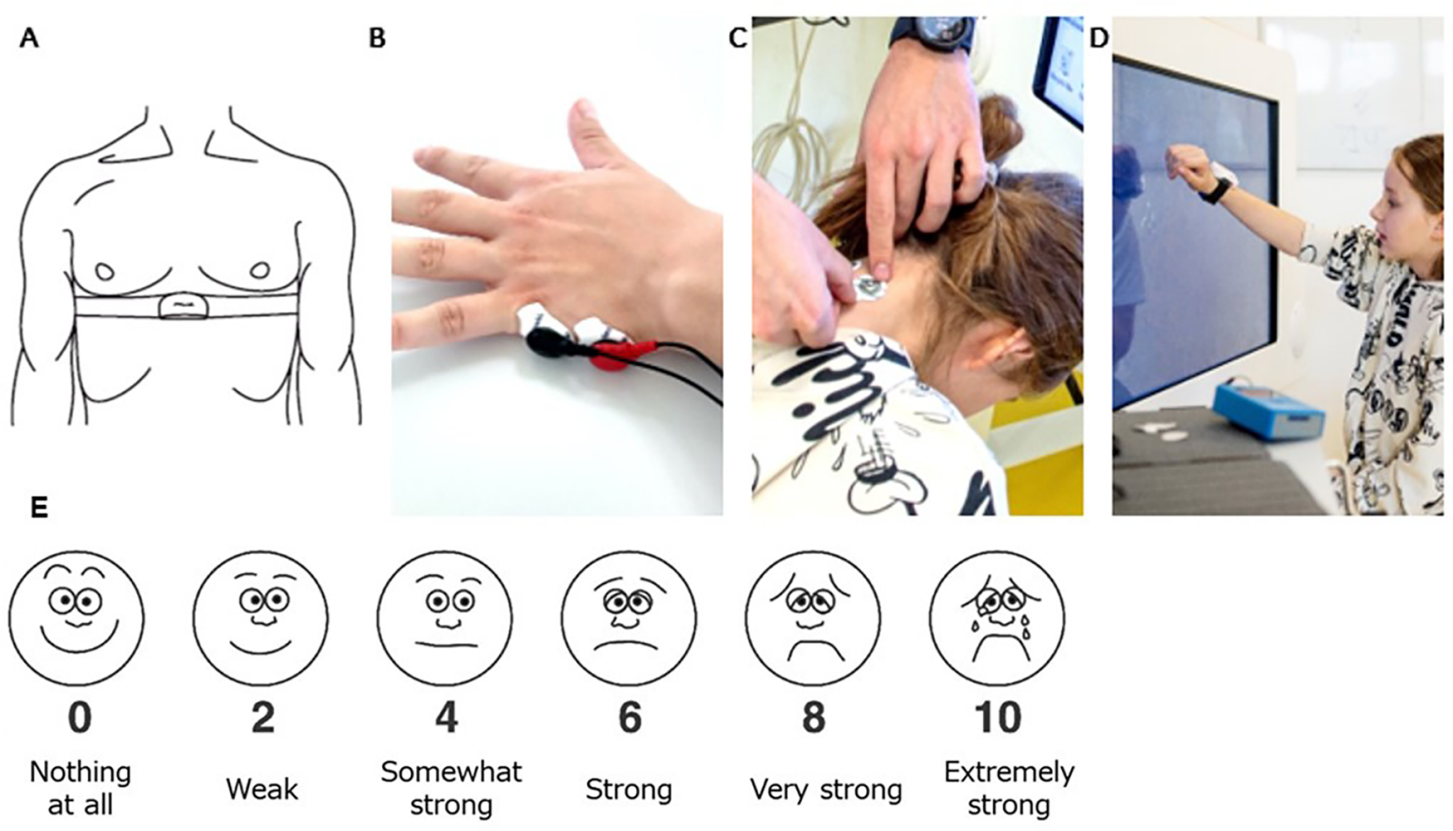
Participant setup. **(A)** A chest strap with a Polar H10 Heart Rate Sensor tied tightly caudally of the pectoral muscles to record the heart rate that is used to calculate the heart rate variability. **(B)** Electrodes positioned on the hypothenar eminence of the non-trained hand, and **(C)** on the neck paramedial below the hairline to measure skin conductance. **(D)** The IMU sensor was placed dorsally around the wrist of the trained hand to measure activity counts. **(E)** Visual Analogue of the Borg Scale from Nashimoto and colleagues (2021). We translated the scale into the German language. The participants were informed that interval numbers (i.e., 1,3,5,7,9) could also be given.

*Heart rate variability* (Figure 3A): We measured heartbeat intervals using a Polar H10 heart rate sensor (Polar, Kempele, Finland) and the Elite HRV application (Asheville, USA) and calculated the HRV. We followed the recommendations of the Task Force of the European Society of Cardiology and the North American Society of Pacing and Electrophysiology (20), which recommend measuring the heartbeat intervals in a sitting position for at least five minutes. We calculated the root mean square of successive differences (RMSSD) from R-to-R intervals using the Kubios HRV software (32). Before calculating the RMSSD, we filtered out the artifacts. This consisted of two steps: (1) selecting a threshold that identified the artifacts and (2) replacing the artifacts with interpolated values using a cubic spline interpolation. We selected a 1% correction threshold, as in measurements including few upper body movements and low VO_2_ Max percentages, heart rate artifacts are not likely to be higher than 1% (33). The 1% correction rate may be the highest possible level for correcting abnormal beats without artificially influencing the data, as Rogers and collaborators (34) observed that artifact corrections of 1%–3% could, although minimally, bias HRV values. Finally, to remove the mathematical bias derived from the non-linear inverse relationship between R-R and heart rate, we normalized the RMSSD data with the average R-R interval signal (35).

*Skin conductance* (Figure 3B,C): We measured SC with the MentalBioScreen K3 device (Porta Bio Screen GmbH, Berlin, Germany) by placing two electrodes on the hypothenar eminence of the non-trained hand and two on the neck. We calculated the mean SC values (µS) for each intensity level for the hand and neck using MATLAB (Version R2022a. The MathWorks Inc. Natick, MA, USA). As SC is known to be affected by temperature and humidity, we tried to keep these equal between measurement days.

*Activity counts per minute* (Figure 3D): We placed a Shimmer3® IMU (Shimmer Research Ltd, Dublin, Ireland) at the wrist of the trained upper limb. We extracted, filtered, resampled, and summed raw 3-dimensional acceleration data using an open-source MATLAB code (36). Afterward, we derived the activity counts per minute (AC/min).

*Movement repetitions per minute:* one rater visually analyzed the video recordings. The rater watched the videos at a lowered speed and counted the participant's movements with a digital counter. A movement was defined as an interaction with the exergame, regardless of whether this was positive or negative for the outcome of the exergame. In the Myro®, a movement was counted every time the participants touched the touch screen. In the ArmeoSping®, a movement was counted every time the participants interacted with the “mouse” (controlled by their arm) in the game. By dividing the total number of movement repetitions by the duration, i.e., eight minutes, we derived the movement repetitions per minute (MOV/min).

*Perceived exertion* (Figure 3E): the participants scored their subjective level of exertion after each intensity level. We provided the visual analog scale of the adapted (0–10) Borg scale, which has been proven to be a valid alternative to the Borg scale in children's and adult's exercise (37, 38). Perceive exertion scales such as the Borg scale can differentiate between intensity and task difficulty levels (39) when the participants are cognitively fit. Due to our inclusion criteria, our participants had to have a certain level of cognition justifying using this scale.

### 2.5 Statistical analysis

We used R Studio (RStudio Inc., Boston, USA) for the statistical analysis. We used descriptive statistics to summarize data and check for normality. In the case of normally distributed data, we performed a one-way repeated measures ANOVA to compare differences between the three intensity levels for each measure. In the case of non-normally distributed data, we used Friedman's test. We set the alpha level at 0.05. When conditions differed significantly, *post-hoc* analyses included paired *t*-tests (normally distributed data) or Wilcoxon signed-rank tests (non-normally distributed data). We corrected for multiple comparisons using the Bonferroni correction.

We analyzed the test-retest reliability of each measure by calculating the ICC. Following Koo and Li's guidelines (40), we selected a two-way mixed effect, absolute agreement, single rater/measurement ICC form, which equates to the ICC (2,1) proposed by Shrout and Fleiss (41). We categorized ICC values under 0.5 as poor reliability, 0.5–0.75 as moderate, 0.75–0.9 as good, and above 0.9 as excellent.

## 3 Results

Twelve children and adolescents (5 females) 8–18 years old (mean ± SD;11.92 ± 3.03 years) were included in the analysis. We could only recruit one participant from the 5-8 years group and we excluded one participant from the 13–18 years group a posteriori because the diagnosis was changed and no longer matched the study's inclusion criteria. Cerebral palsy (4), stroke (2), and brain tumor (2) accounted for most of the diagnoses (see Table 1). We obtained complete data from 9 out of the 12 participants. One participant showed extreme artifacts in the HRV of the second session, and two had technical problems with the sensors in one of the sessions. One participant informed us that the “very difficult” intensity level from day one was too demanding for him and needed to be adapted. We did not include the data derived from this intensity level in the reliability analysis.

**Table 1:** Characteristics of the participants.

ID	Gender	Age (years)	Diagnosis	Trained arm	Device	Therapy goal
1	Female	11	Neurapraxia N. radialis (Polytrauma)	Left	Armeo Spring	Increase the range of motion and general activity of the affected extremity
2	Male	10	Stroke	Right	Armeo Spring	Improve motor planning, problem solving, while reducing learned non-use of the affected extremity
3	Male	18	Brain Tumor	Left	Myro	Increase muscular endurance, movement velocity, and precision
4	Female	14	Brain Tumor	Right	Myro	Increase muscular endurance
5	Female	15	Traumatic Brain Injury	Right	Myro	Increase muscular endurance
6	Female	14	Unilateral Cerebral Palsy	Right	Armeo Spring	Increase the use of the affected extremity
7	Male	10	Muscular Dystrophy	Left	Myro	Avoid the decrease of strength and muscular endurance
8	Female	9	Bilateral Cerebral Palsy	Right	Myro	Reduce learned non-use of the affected extremity
9	Male	14	Stroke	Left	Myro	Reduce learned non-use of the affected extremity
10	Male	9	Meningoencephalitis	Left	Myro	Increase strength and muscular endurance
11	Male	8	Unilateral Cerebral Palsy	Left	Myro	Reduce learned non-use of the affected extremity
12	Male	11	Unilateral Cerebral Palsy	Right	Myro	Reduce learned non-use of the affected extremity

### 3.1 Comparison among intensity levels

**Figure 4:**
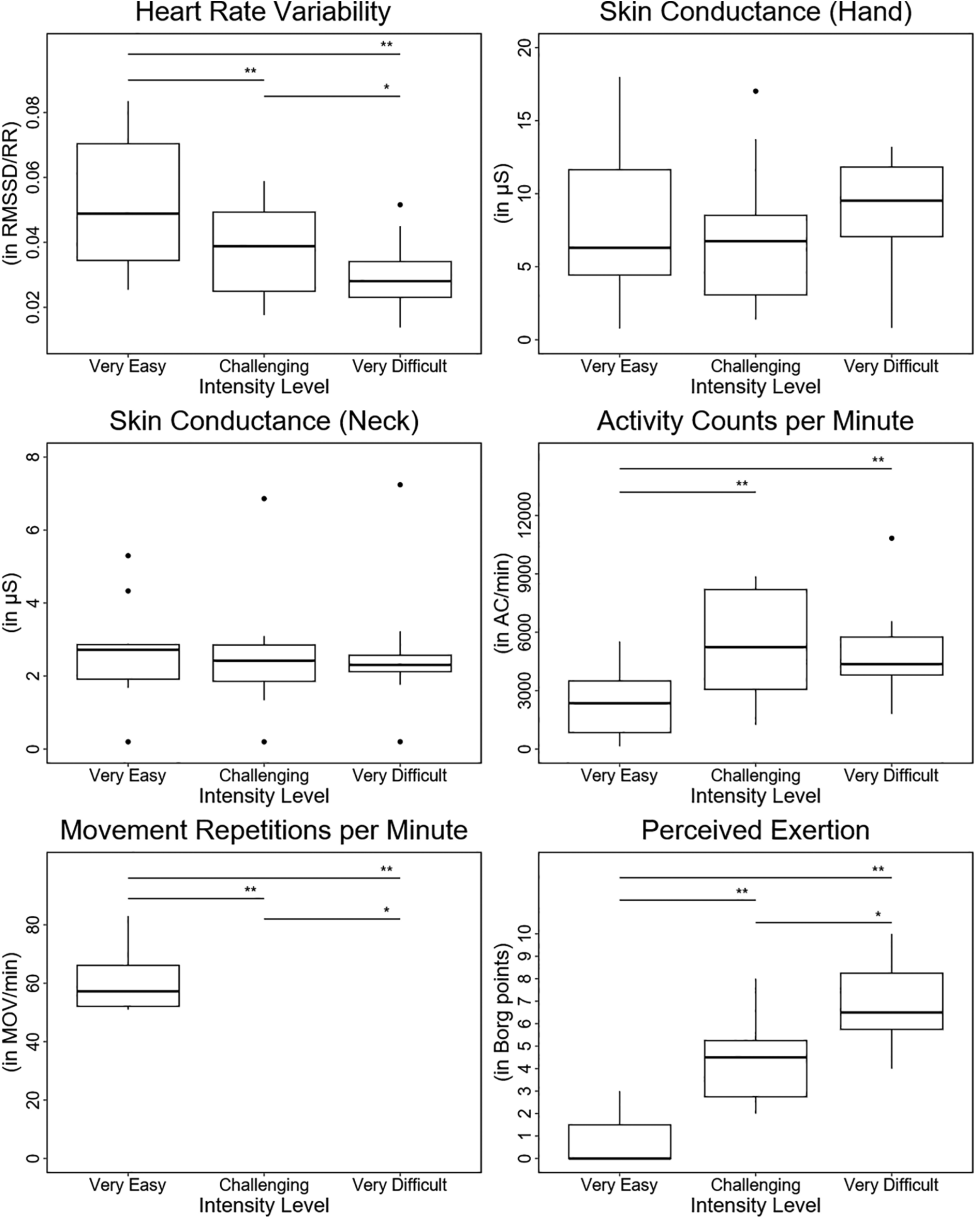
Results. Boxplots depicting the results of each outcome measure for each intensity level. In addition, the statistical significance of the pos hoc test for each comparison between intensity levels is displayed. *: *p* < 0.05; **: *p* < 0.01; ***: *p* < 0.001.

Heart rate variability showed statistically significant differences between the three therapy intensity levels, with higher scores in the “very easy” than in the “challenging” intensity level and lower scores in the “very difficult” than in the “challenging” intensity level (see Table 2 and Figure 4). Skin conductance did not show statistically significant differences between any of the intensity levels.

**Table 2:** Responses of the intensity measures.

Measure (unit)	N	Median (IQR[Q1,Q3)			Primary test	*Post-hoc* tests		
		Very easy	Challenging	Very difficult	*P*-value	Very easy—challenging *P*-value	Very easy—very difficult *P*-value	Challenging—very difficult *p*-value
HRV (RMSSD/RR)	11	0.038 [0.033, 0.069]	0.035 [0.025, 0.048]	0.028 [0.024, 0.033]	**0.001**	**0.005**	**0.003**	**0.029**
SC Hand (µS)	12	6.30 [4.43, 11.64]	6.75 [3.07, 8.51]	9.52 [7.06, 11.83]	0.27	–	–	–
SC Neck (µS)	12	2.72 [1.92, 2.86]	2.42 [1.86, 2.85]	2.3 [2.11, 2.57]	0.64^a^	–	–	–
AC/min (#)	10	2359.3 [851.7, 3500.4]	5239.1 [3068.3, 8191]	4368.3 [3803.6, 5757.1]	**<0.001**	**0.002**	**0.007**	0.1
MOV/min (#)	12	8 [7, 12]	29 [24, 30]	53 [28, 69]	**<0.001** ^a^	**0.003**	**0.003**	**0.02**
Perceived Exertion (Borg 1–10)	12	0 [0, 1.5]	5 [3, 5]	7 [6, 8]	**<0.001** ^a^	**0.01**	**0.007**	**0.017**

*P*-values from the *Post-Hoc* tests are Bonferroni corrected. Bold values represent statistical significance.

N, number of patients included in the statistical analysis; IQR, inter quartile range; Q1, 1st quartile; Q3, 3rd quartile; ^a^, not normally distributed; HRV, heart rate variability; RMSSD, root mean squared of successive differences; SC, skin conductance; AC/min, activity counts per minute; MOV/min, movement repetitions per minute.

The activity counts per minute were significantly higher in the “challenging” and “very difficult” intensity levels than in the “very easy” intensity level. Furthermore, despite not being statistically significant, the AC/min tended to be lower in the “very difficult” intensity level than in the “challenging” intensity level.

The movement repetitions per minute and the Borg scale exhibited statistically significant differences between the three therapy intensity levels, showing lower scores in the “very easy” than in the “challenging” intensity level and higher scores in the “very difficult” than in the “challenging” intensity level.

### 3.2 Test-retest reliability (ICC)

Generally, test-retest reliability ranged from poor (ICC = 0.29) for the SC measured on the hand in the “very difficult” intensity level to excellent (ICC = 0.98) for the MOV/min for the “very difficult” intensity level (Table 3). Heart rate variability showed moderate to good reliability (ICC = 0.75 for the “very easy” intensity level, 0.62 for the “challenging” intensity level, and 0.79 for the “very difficult” intensity level), while SC showed poor to moderate reliability for the hand (ICC = 0.67, 0.44, and 0.29) and moderate to good reliability for the neck (ICC = 0.73, 0.83, and 0.84). Activity counts per minute exhibited good to excellent reliability (ICC = 0.92, 0.94, and 0.89), and MOV/min good to excellent reliability (ICC = 0.92, 0.89, and 0.98). The Borg scale showed moderate to good reliability (ICC = 0.80, 0.70, and 0.85).

**Table 3:** Reliability of the intensity measures.

Measure	N	ICC (95% CI [LL,UL)					
		Very easy	*P*-value	Challenging	*P*-value	Very difficult	*P*-value
HRV	10	0.75 [0.32, 0.92]	**0.002**	0.62 [0.08, 0.88]	**0.013**	0.79 [0.41, 0.94]	**<0.001**
SC Hand	11	0.67 [0.19, 0.90]	**0.006**	0.44 [−0.16, 0.81]	0.076	0.29 [−0.23, 0.73]	0.15
SC Neck	11	0.73 [0.27, 0.92]	**0.002**	0.83 [0.27, 0.96]	**<0.001**	0.84 [0.08, 0.97]	**<0.001**
AC/min	9	0.92 [0.75, 0.97]	**<0.001**	0.94 [0.81, 0.98]	**<0.001**	0.89 [0.69, 0.97]	**<0.001**
MOV/min	11	0.92 [0.77, 0.98]	**<0.001**	0.89 [0.67, 0.97]	**<0.001**	0.98 [0.89, 1]	**<0.001**
Perceived exertion	11	0.80 [0.45, 0.94]	**<0.001**	0.70 [0.26, 0.90]	**0.004**	0.85 [0.58, 0.95]	**<0.001**

Bold values represent statistical significance.

N, number of patients included in the statistical analysis; ICC, intraclass correlation coefficient; CI, confidence interval; LL, lower limit; UL, upper limit; HRV, heart rate variability; SC, skin conductance; AC/min, activity counts per minute; MOV/min, movement repetitions per minute.

## 4 Discussion

This study investigated for the first time the responses of several candidate intensity measures to three different intensity levels of upper limb robotic exercises in children with neurological diagnoses. In addition, we investigated the test-retest reliability on two independent test occasions. According to the COSMIN guidelines (42), the number of participants should have been higher for an accurate psychometric evaluation. Therefore, we discuss the potential of the measures to reflect intensity rather than their validity and refer to the reliability as preliminary. The main results were the following: first, the HRV, MOV/min, and perceived exertion differed between each intensity level. Second, AC/min differed between “challenging” and “very easy” but not between “challenging” and “very difficult.” Third, SC responses did not differ between any of the three intensity levels. Fourth, the preliminary reliability proved acceptable, i.e., ICC ≥ 0.75, for all intensity levels of AC/min and MOV/min.

### 4.1 Responses

Heart rate variability was lower at higher therapy intensity levels, indicating the need for higher sympathetic activation to match the higher demands of the more complex tasks. Similar changes in HRV profile can be found in studies analyzing the effects of the increments in mental task difficulty (17, 18) and motor intensity (22, 23) in healthy adults. However, this is the first study showing such results in children with a lesioned CNS, who might experience autonomic dysregulation (43, 44) and reduced HRV and HRV adaptability (45).

Skin conductance did not respond to any changes in exercise intensity. Although skin conductance has several times been shown to react to changes in task difficulty during video gaming (18, 19, 25, 26), these tasks had reduced or inexistent motor load. In our exergames, participants had to perform high amounts of motor activity in addition to the mental load. The increased body activity may have led to increased activity of the sweat glands, thus confounding the results by masking the pure CNS activation. Furthermore, although SC responses in the neck have been correlated to the reactions of the hypothenar eminence (46) and could be a better option for bimanual activities, these are not commonly used. Our data showed different responses for both placements. For the hypothenar eminence, SC was slightly lower in the “very easy” intensity level than in the “challenging” intensity level and higher in the “very difficult” intensity level than in the “challenging” intensity level. In contrast, for the neck, SC showed slightly lower values as the intensity levels increased. This mismatch between the skin conductance measured on the hand and the neck suggests that these placements are not interchangeable.

Activity counts per minute could not differentiate between the “challenging” and the “very difficult” intensity levels. Moreover, although statistically insignificant, the “challenging” intensity level showed higher mean counts per minute than the “very difficult” intensity level. This was surprising initially, as we expected activity counts to increase linearly with the number of movement repetitions (47). However, when comparing the movements performed in the “challenging” and “very difficult” intensity levels, we realized that, in some cases, the characteristics of the movements differed greatly. While the “challenging” intensity levels required whole arm movements with large range of motions at medium to moderate speeds, some “very difficult” intensity levels required shorter but faster movements. The video footage showed that patients often performed these fast movements by repeatedly tapping at the same or nearby location on the screen. For these interactions, patients used wrist flexion-extension and abduction-adduction movements. As we placed the IMU on the wrist, this isolated hand activity might not have been adequately quantified. Hence, IMU placement and exercise characteristics may bias the responses.

The number of movement repetitions per minute was higher at higher therapy intensity levels, which matches our hypothesis. Therapy intensity in neurorehabilitative interventions is usually equated with the number of movement repetitions (48), and, therefore, therapists might be predisposed to increase the number of repetitions when told to increase therapy intensity. However, when analyzing the video recordings, we realized that therapists also used other ways to increase therapy intensity. In the case of patient ID2 (see Table 1), the therapist increased the therapy intensity by increasing the mental load of the task. In general, patients can have treatment goals that focus more on mental rather than motor demands. ID2, for example, had the main therapy goal of improving motor planning and problem-solving. This participant performed fewer repetitions in the “very difficult” intensity level than in the “challenging” intensity level (203 vs. 247) despite perceiving the “very difficult” intensity level as more difficult compared to the “challenging” one (Borg scale 5 vs. 2). ID2 also showed a lower HRV in the “very difficult” compared to the “challenging” intensity level (0.052 vs. 0.059) (see Supplementary Material S2). The results suggest that quantifying therapy intensity only by movement repetitions per time unit neglects other important aspects.

Perceived exertion, as measured by the Borg scale, differed between the three intensity categories. Robert and collaborators (49) analyzed the responses of the Borg scale to different intensity levels of an active video game in ten children with cerebral palsy. They found only near-significant differences, making this the first study in which the Borg scale was able to distinguish significantly between upper limb exercise intensity levels in children with neurological diagnoses.

### 4.2 Reliability

We analyzed ICC scores to assess the test-retest reliability of the measures. Koo and Li (40) suggested that, instead of the ICC scores, the 95% confidence interval should be taken into account to interpret the test-retest reliability results correctly. However, these recommendations were made based on the assumption of having a sample size of at least 30 participants, which is the minimal sample size expected for psychometric studies. Given that our study is exploratory and has a small sample size, and considering the impact sample size has on confidence intervals, we will focus solely on the ICC scores during the discussion, referring to the reliability as preliminary.

Heart rate variability exhibited moderate to good preliminary test-retest reliability. These results are in line with other studies in healthy adult populations at rest and during light activities (50, 51) and neurological populations at rest (52, 53), which reported ICC values around 0.70.

Skin conductance exhibited poor to moderate preliminary reliability for the hand and moderate to good reliability for the neck. Skin conductance is known to show different test-retest reliabilities in various experimental situations (54, 55). Although the experimental context was different, Cooper and colleagues (27) showed similar ICC values for the SC measured on the hypothenar eminence of the hand.

Activity counts per minute exhibited good to excellent ICC scores for all intensity levels. These results are in line with the literature, where it has been shown that IMU-based data have good test-retest reliability, regardless of the purpose for which they are used (28, 56).

Movement repetitions per minute exhibited ICC values ranging from good to excellent. This suggests that the underlying algorithm controlling the course of the game resulted in a consistent number of movement repetitions for each difficulty level and that the children played the game similarly, ensuring that the procedure was comparable for both days.

Last, the Borg scale exhibited moderate to good preliminary reliability, which is consistent with the literature (29, 57). Using a numerical Borg scale of 1–10, the authors found ICC values of 0.78 and 0.92, respectively. Van der Zwaard and colleagues analyzed in addition the reliability of a visual analog scale similar to the one we used and reported an ICC value of 0.74.

### 4.3 Practical use and potential

Heart rate variability responded to the different intensity levels as hypothesized, showed moderate to good preliminary reliability, and was easy to use. Initially, to account for the between-patients variability, we tried to normalize HRV to the mean value obtained in the rest condition. Following the HRV results, which decreased with increasing intensity levels, we expected higher HRV levels at rest than in the “very easy” intensity level. However, while some patients showed the highest HRV level at rest, others showed lower HRV levels, sometimes even lower than those measured during the “very difficult” intensity level. While we assume that these values resulted from the patient's nervousness, it shows that it might be challenging for this group of children with neurological diagnoses to get a reliable HRV measurement during rest. Another option might be to normalize HRV to where the patient works at a maximum level. A normalization procedure towards the maximal capacity is standard for endurance training [i.e., where individuals train at a certain percentage of the maximal heart rate (58)] and strength training [i.e., where individuals train at a certain percentage of the one repetition maximum (59)]. Perhaps a maximal test could help account for between-patient HRV differences and enable us to have patients perform at relative percentages of the minimum HRV obtained during maximal capacity. This would allow us to adapt and individualize therapies better. However, this method has never been used, and its practicability remains unknown.

Skin conductance did not respond to the different intensity levels and exhibited insufficient reliability when measured at the hand. In addition, it is influenced by changes in temperature and humidity, two factors that cannot be easily controlled for in a therapy setting. Our findings suggest that SC may not be a good intensity measure for clinical practice.

Activity counts per minute responded partially to the intensity levels, exhibited acceptable reliability, and demonstrated to be an easy-to-use measure. Activity counts per minute is already a better representative of the patient's activity than raw activity counts, as it accounts for the temporal component. However, AC/min is an absolute measure, and in its current form, it does not reflect how hard a patient works, relative to their capacity, during a therapy session. For example, when being pushed to their limits for one minute, patients with mild upper-limb impairments may be able to open and close ten buttons of a jacket, a recurring exercise during conventional occupation therapy, or touch the screen 20 times. In contrast, a patient with severe impairments may only be able to do one-tenth of the repetitions in the same amount of time. By measuring intensity with absolute measures, the information about the patient's capabilities is lost; hence, it might appear that the patient with fewer impairments trains harder. However, when considering the capabilities, both patients train at their maximum. For this reason, relative intensity measures are needed. To use AC/min as a relative intensity measure and personalize it for therapy, we would need additional information, such as a maximum capacity value, e.g., the maximal number of counts that a patient can perform for a given exercise and time unit. This would allow us to calculate the intensity level at which the patient exercises relative to their capabilities.

Movement repetitions per minute responded to the different intensity levels and showed excellent preliminary reliability. Although they were easy to record, the analysis was very time-consuming. For instance, analyzing the “very difficult” intensity levels required up to 30 min. Furthermore, our tasks comprised discrete movements, and we defined one movement as one successful interaction with the game. However, many other therapies may involve more complex indiscrete or non-cyclic movements, making it difficult to define and quantify the number of repetitions. Similarly to AC/min, MOV/min in its current form is also an absolute intensity measure. Consequently, a maximal capacity value would be needed to reflect the intensity level at which the patient works relative to their capabilities.

Finally, perceived exertion measured with the Borg scale responded to the different intensity levels, showed good preliminary reliability, and was easy to use. In addition, as it is already relative to a maximum, it provides information about how hard the patient works during therapy, i.e., intensity, and can become an intensity measure in pediatric neurorehabilitation. However, our inclusion criteria required children to understand and respond well to questions. Children with less cognitive ability may not be able to rate their perceived exertion reliably. Furthermore, the Borg scale implies listening and responding to a question. If we would like to use an intensity measure to continuously monitor the intensity of the therapy during the session and adapt the intensity as needed, self-reported perceived exertion scales may not be the best option. From our experience, repeatedly rating the perceived exertion can be tedious and lower patient engagement, disrupting the flow of the session, and compromising the therapy. Therefore, although the Borg scale is a good measure to get a global impression of the overall therapy intensity level at the end of a session, other measures may hold a greater potential to measure therapy intensity in real time.

## 5 Methodological considerations and limitations

We ensured that the experimental setting stayed as close as possible to the clinical practice. The therapist tailored the intensity levels individually towards the patient's capacity and therapeutic goals and decided how to change intensity meaningfully. Furthermore, we used off-the-shelf therapeutic devices with exergames. While we considered this initially a strength of our approach, it also resulted in various limitations. First, we did not account for the influence of the therapy goal beforehand. Most rehabilitation technologies have been designed to provide repetitive movements to support patients in regaining motor function. However, as we noticed in participant ID2 (see Table 1), therapists can increase intensity by adjusting the complexity of the tasks, e.g., by increasing the mental load or complexity of the movements. Increasing the mental load might have affected our intensity measures differently, and further research is needed to provide a better insight into how various candidate measures would respond to different levels of motor or mental intensity. Second, one could argue that the intensity levels (“very easy”, “challenging”, and “very difficult”) were somewhat subjective, as we could not objectively assess the success rate of each intensity level. While we acknowledge this limitation, we think the therapist did this rather well, as the Borg results were well in line with the intensity levels: median value for the intensity level “very easy”: 0 (“nothing at all”), “challenging”: 5 (“somewhat strong” to “strong”), and for the “very difficult” intensity level: 7 (“strong” to “very strong”). Third, off-the-shelf games allow a particular variation in intensity levels, but it was difficult to standardize the games for several intensity levels or adapt them objectively to the patient's capabilities. Furthermore, therapists lacked specific control over various features to keep other variables comparable between intensity levels. For example, patients could use strategies that affected other movement components (e.g., some increased the number of movements while reducing the range of motion with increasing difficulty level). Moreover, the games did not allow us to increase only motor or mental load, which could have caused interference and might have affected the behavior of some of the intensity measures. Fourth, although we consider that letting the therapists decide how to adapt the intensity is more appropriate to account for patient heterogeneity and goals and relates closer to the clinical practice than controlling for it, we agree that this might have impacted the responses and that a more controlled environment may be better to understand the responses. For these reasons, we are developing customized exergames that would help us overcome these limitations by allowing us to better standardize and adapt the intensity levels and load types.

Furthermore, we could only include one participant between 5 and 8 years old, which limits the generalizability of our findings. Our inclusion criteria required children to understand simple instructions and comply well. This made recruiting the youngest participants difficult, as most patients in our rehabilitation clinic show a delay in their cognitive development.

In addition, we did not analyze the 95% confidence intervals of the ICC values, as suggested by Koo and Li (40). We consider that analyzing the confidence intervals in studies with small sample sizes reflects the sample size more than the reliability of the measures, potentially leading to misinterpretation of the results. Nevertheless, we agree that in future confirmatory studies with larger sample sizes, the 95% confidence interval should be considered.

Finally, it must be reiterated that these preliminary results are specific to the population and therapy type, i.e., technology-assisted therapy. To confirm whether these intensity measures could be applied to other neurological patient groups and a broader range of neurorehabilitative therapeutic approaches, confirmatory studies are needed to assess the validity of these measures.

## 6 Conclusion

Although intensity appears to be one of the most critical factors affecting rehabilitative outcomes, there is still no objective and universal way of measuring it when considering its multidimensionality. We showed that HRV, MOV/min, and perceived exertion responded to different intensity levels and showed preliminary reliability when investigated on two separate days. This highlights their potential for becoming intensity measures for upper limb neurorehabilitation. Particularly, the HRV proved practical because of its potential for application in young patients and clinical practice. However, as each of the measures reflects different aspects of intensity, a combination of measures may be needed to capture the multidimensionality of intensity. In addition, studies with larger sample sizes are needed to gain a deeper understanding about the responses and to confirm these results.

Improving the assessment of therapy intensity is urgently needed to show how different interventional parameters affect the effectiveness of neurorehabilitative interventions. While we performed our experimental approach in line with the clinical application of upper limb technology-assisted treatment in young patients, we noted several limitations and recognized that our current approach did not capture the multidimensional nature of therapy intensity. We have started developing customized exergames that will allow us to better standardize and adapt our approach. These experiments would elaborate our current findings and improve our understanding of how the various candidate measures respond to different motor or mental intensity levels.

## Data Availability

The raw data supporting the conclusions of this article will be made available by the authors, without undue reservation.

## Supplementary material

Supplementary Material S1 Games and levels. Explanatory list of the games the patients played in each intensity level.

Supplementary Material S2 Effects of therapy goal. Depicts the results of each outcome measure for each intensity level, with special focus on therapy goals. Each line depicts one patient, being the black lines patients with motor goals and the red line the patient who had the mental goal.
