# Supplementary material for: Responses of several measures to different intensity levels of upper limb exergames in children with neurological diagnoses: a pilot study
Source: Front Rehabil Sci. 2024 Oct 23;5:1405304. doi: 10.3389/fresc.2024.1405304 (PMC11538011; doi:10.3389/fresc.2024.1405304)
Supplement: Supplementary Material S1 — Games and levels. Explanatory list of the games the patients played in each intensity level. [file Table1.docx]

| ID | Device | Therapy Goal | Game (level) | | |
| --- | --- | --- | --- | --- | --- |
|  | | | Very easy | Challenging | Very difficult |
| 1 | Armeo Spring | Motor | ArmeoControl 1.24  Rain Cup (1) | Zombies vs. Plants (Adventure 2x10) | Zombies vs. Plants (Wall-Nut Bowling) |
| 2 | Armeo Spring | Mental | ArmeoControl 1.24  Rain Cup (1) | Zombies vs. Plants (Zombie Boss) | Zombies vs. Plants (Slot Machine) |
| 3 | Myro | Motor | Zombies vs. Plants (Adventure 1x1) | Zombies vs. Plants (Wall-Nut Bowling) | Zombies vs. Plants (Whack-a-Zombie) |
| 4 | Myro | Motor | TyroS 4.2.1  Apple Farmer (1)  Elevator (1) | Zombies vs. Plants (Adventure 1x5) | Zombies vs. Plants (Wall-Nut Bowling) |
| 5 | Myro | Motor | TyroS 4.2.1  Apple Farmer (1)  Elevator (1) | Zombies vs. Plants (Wall-Nut Bowling) | Zombies vs. Plants (Whack-a-Zombie) |
| 6 | Armeo Spring | Motor | ArmeoControl 1.24  Rain Cup (1) | Zombies vs. Plants (Adventure 1x1) | Zombies vs. Plants (Wall-Nut Bowling) |
| 7 | Myro | Motor | TyroS 4.2.1  Apple Farmer (1)  Elevator (1) | Zombies vs. Plants (Wall-Nut Bowling) | Zombies vs. Plants (Whack-a-Zombie) |
| 8 | Myro | Motor | TyroS 4.2.1  Apple Farmer (1)  Elevator (1) | Zombies vs. Plants (Adventure 1x5) | Zombies vs. Plants (Whack-a-Zombie) |
| 9 | Myro | Motor | TyroS 4.2.1  Apple Farmer (1)  Elevator (1) | Zombies vs. Plants (Adventure 1x5) | Zombies vs. Plants (Wall-Nut Bowling) |
| 10 | Myro | Motor | TyroS 4.2.1  Apple Farmer (1)  Elevator (1) | Zombies vs. Plants (Wall-Nut Bowling) | Zombies vs. Plants (Whack-a-Zombie) |
| 11 | Myro | Motor | TyroS 4.2.1  Apple Farmer (1)  Elevator (1) | Zombies vs. Plants (Wall-Nut Bowling) | Zombies vs. Plants (Whack-a-Zombie) |
| 12 | Myro | Motor | TyroS 4.2.1  Apple Farmer (1)  Elevator (1) | Zombies vs. Plants (Wall-Nut Bowling) | Zombies vs. Plants (Whack-a-Zombie) |

| Game | Screenshot | Description |
| --- | --- | --- |
| ArmeoControl 1.24  Rain Cup (1) | 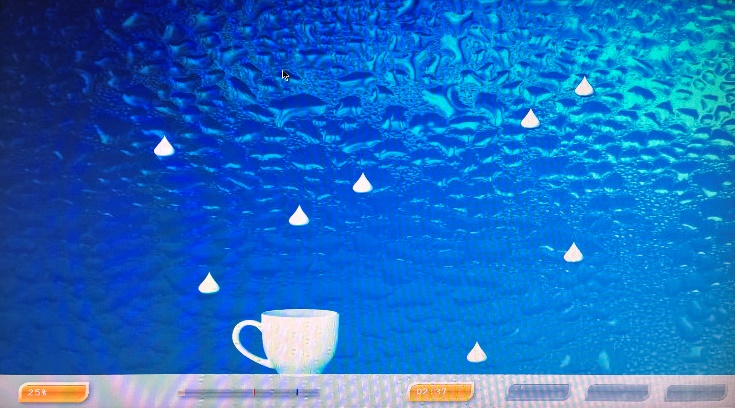 | The aim of the game is to catch raindrops that descend from the clouds with a cup. The participant controls the cup with their own arm movement. |
| TyroS 4.2.1  Apple Farmer (1) | 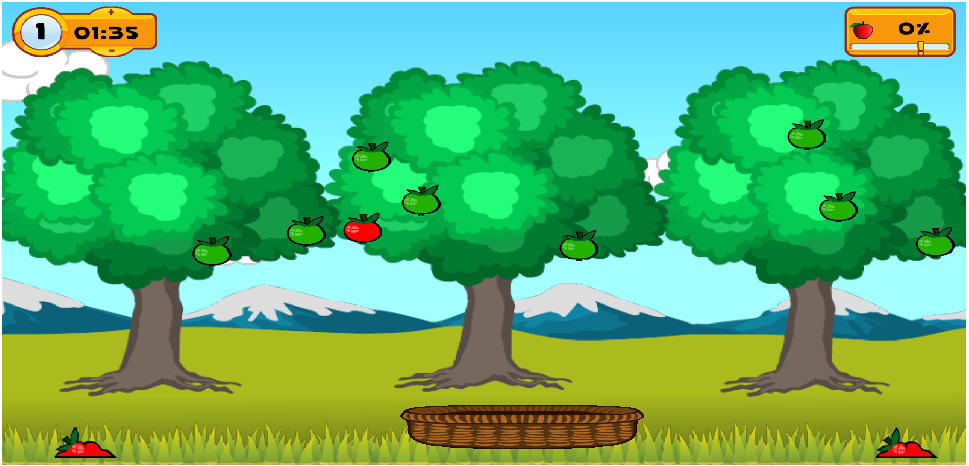 | The aim of the game is to catch apples that fall from three different trees. The participant controls a basket by tipping on the touch screen. |
| TyroS 4.2.1  Elevator (1) | 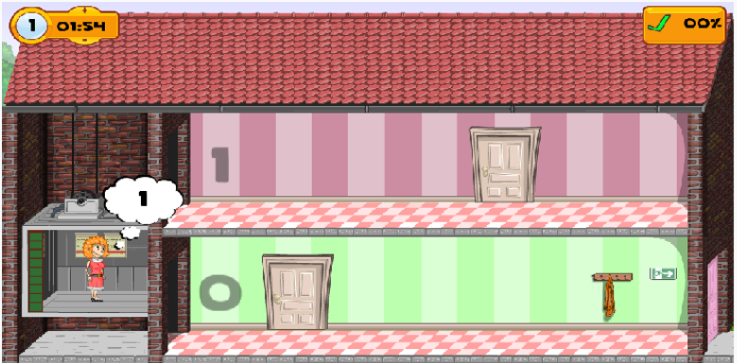 | The aim of the game is to actuate a broken elevator to help people go up and down to the desired floor. The participant controls the elevator by tipping on the touch screen. |
| TyroS 4.2.1  Firefighter (1) | 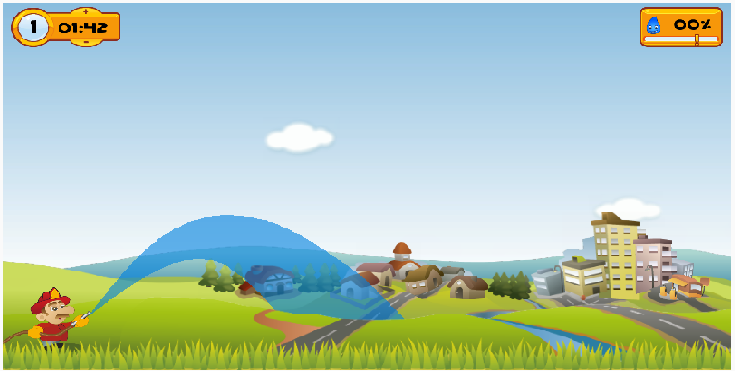 | The aim of the game is to turn a burning fire off to prevent houses from burning. The participant controls the water hose by tipping on the touch screen. |
| Zombies vs. Plants (Adventure xxx) | 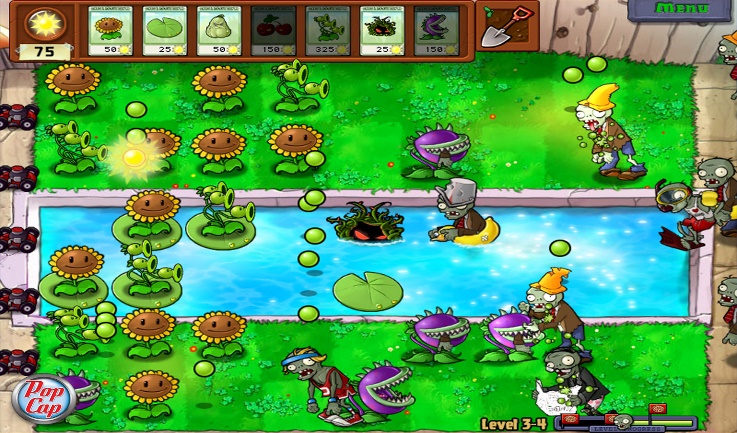 | In this tower defense video game, the player aims to prevent zombies from entering a house through the lawn. To defend the house, the player can use different plants that act against the zombies. To plant the plants, the player must collect suns falling from the sky throughout the screen. The game requires fast movements and strategic thinking to save the house from the zombies. At the end of nearly every level, the player collects a new type of plant to use in subsequent levels. With the new levels, new zombies appear with new and more dangerous characteristics. As the levels go on, the game becomes increasingly challenging concerning the motor load, i.e., the player needs to move faster, and the mental load, i.e., the player needs to process more information and make more decisions. |
| Zombies vs. Plants (Wall-Nut Bowling) | 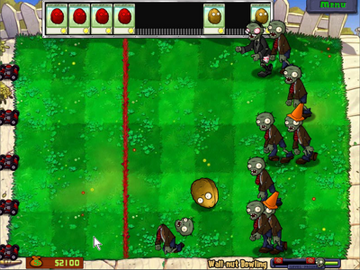 | In this tower defense video game, the player aims to prevent zombies from entering a house through the lawn. To defend the house, the player can use walnuts as if they were bowling balls to throw them at the zombies. The game requires great amounts of movement and some tactics. |
| Zombies vs. Plants (Whack-a-Zombie) | 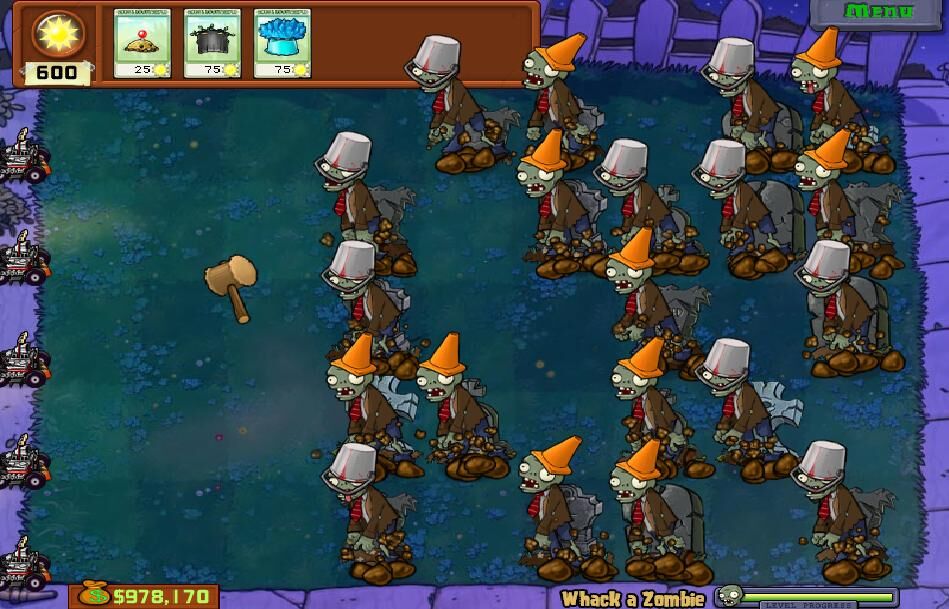 | In this tower defense video game, the player aims to prevent zombies from entering a house through the lawn. To defend the house, the player can use a wooden hammer to hit the zombies. The zombies appear at a very high speed and frequency; therefore, the participants must move very fast to complete the level successfully. |
| Zombies vs. Plants (Zombie Boss) | 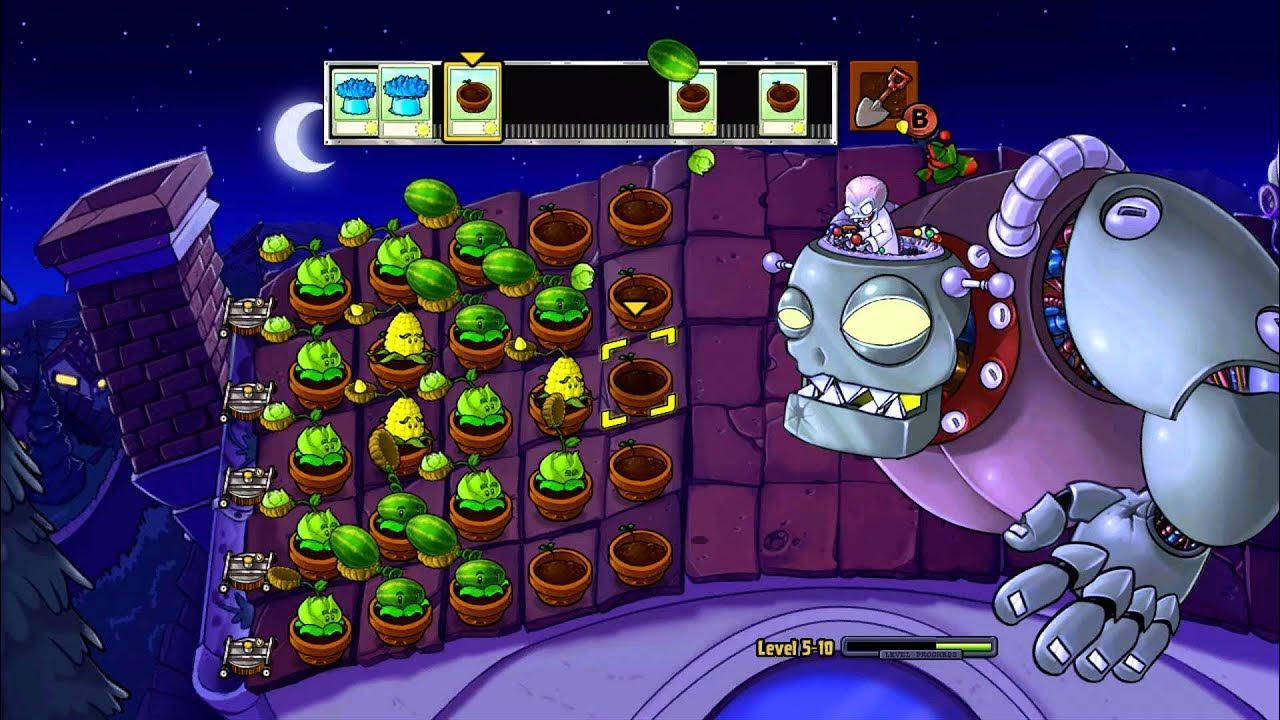 | This is the very last level of the adventure modality. In this case, to defend the house against the zombie boss you do not need to collect suns to plant the plants, but instead, you get automatically get the plants from the game. By doing so, the motor component is slightly decreased. However, the mental component increase, as you are given the plants, without the opportunity of selecting the ones that are more adequate for each moments, which makes the decision making and strategic thinking much more important. |
| Zombies vs. Plants (Slot Machine) | 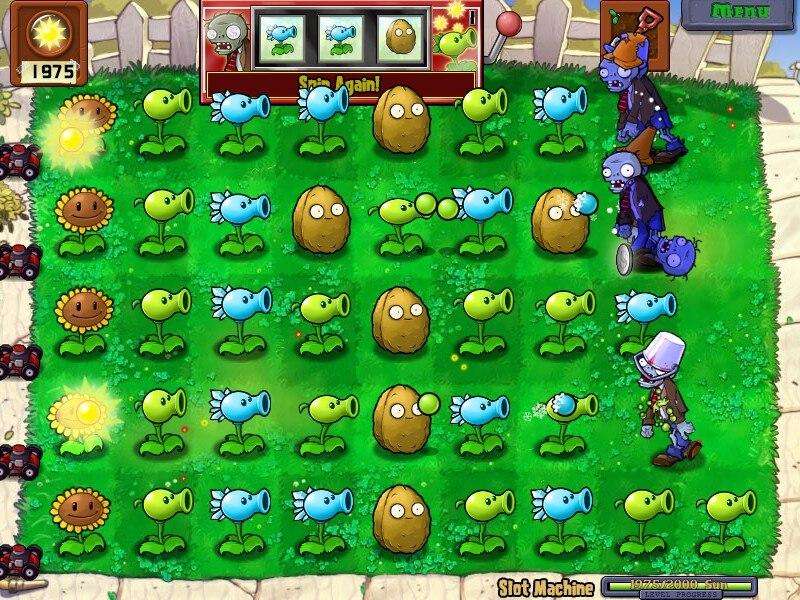 | In this tower defense video game, the player aims to prevent zombies from entering a house through the lawn. To defend the house, the player can use different plants that act against the zombies. To plant the plants, the player must collect suns that fall from the sky, which are then used to activate a slot machine. The slot machine provides the plants that the player must plant to defend the house. The lack of ability to select the plants needed, and the uncertainty of whether the slot machine will provide any plant at all makes the decision making and strategic thinking much more important, increasing the mental load. |
