# Supplementary figures and images for: Responses of several measures to different intensity levels of upper limb exergames in children with neurological diagnoses: a pilot study
Source: Front Rehabil Sci. 2024 Oct 23;5:1405304. doi: 10.3389/fresc.2024.1405304 (PMC11538011; doi:10.3389/fresc.2024.1405304)

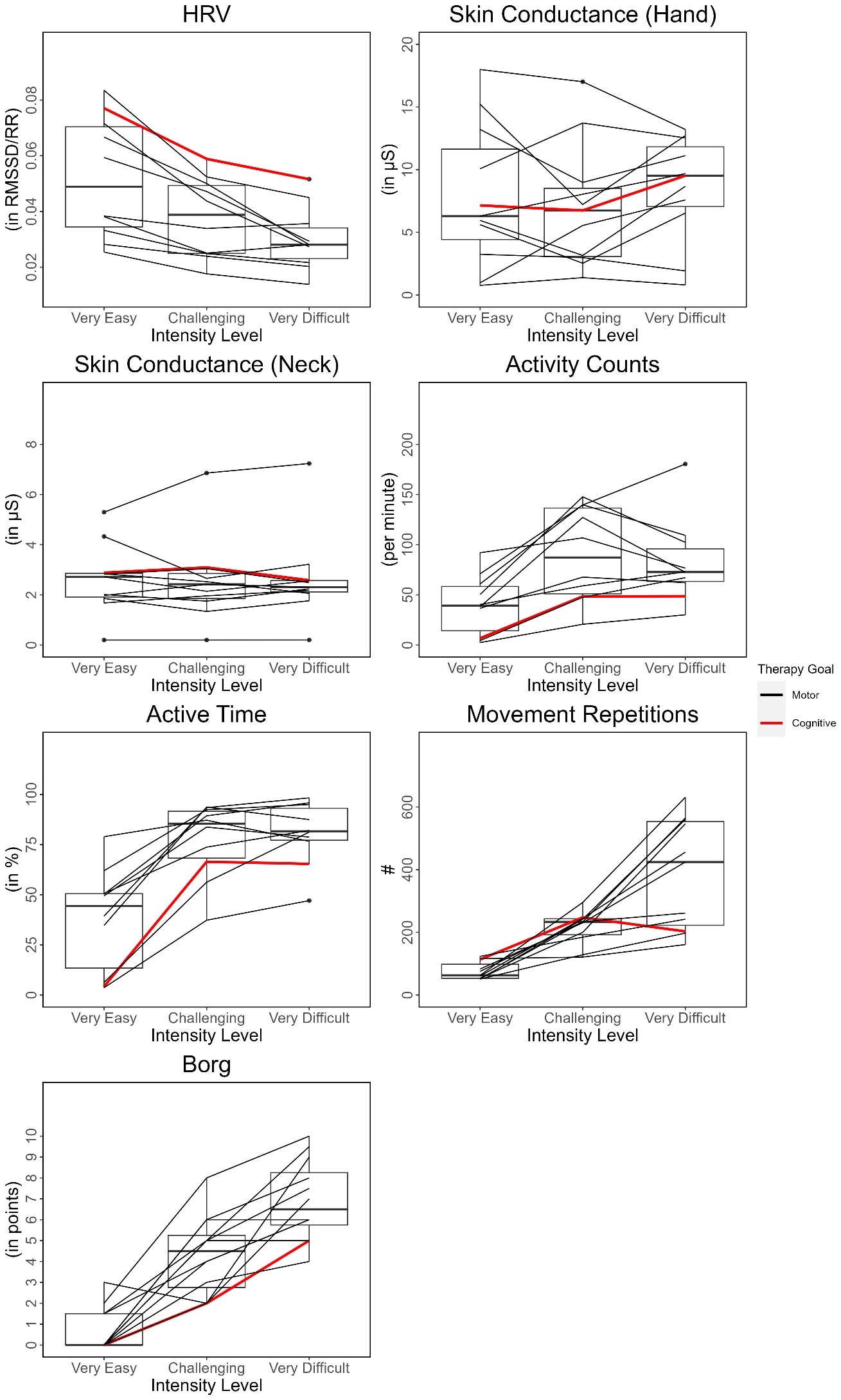

Supplement: Supplementary Material S2 — Effects of therapy goal. Depicts the results of each outcome measure for each intensity level, with special focus on therapy goals. Each line depicts one patient, being the black lines patients with motor goals and the red line the patient who had the mental goal. [file Datasheet1.docx]
